# Genetic landscape of Romanian PPGLs

**DOI:** 10.1111/jcmm.70204

**Published:** 2024-12-13

**Authors:** Sofia‐Maria Lider‐Burciulescu, Monica Gheorghiu, Elena Braha, Laura Semonia Stanescu, Attila Patocs, Corin Badiu

**Affiliations:** ^1^ “Ana Aslan”, National Institute of Geriatrics and Gerontology Bucharest Romania; ^2^ “Carol Davila” University of Medicine and Pharmacy Bucharest Romania; ^3^ “CI Parhon” National Institute of Endocrinology Bucharest Romania; ^4^ Semmelweis University Budapest Hungary

**Keywords:** founder effect, genetics, hereditary, paraganglioma, pheochromocytoma, sporadic

## Abstract

Pheochromocytomas and paragangliomas (PPGLs) are rare neuroendocrine tumours that originate from chromaffin cells and occur in the adrenal medulla and in the sympathetic or parasympathetic ganglia. Nearly 70% of PPGLs result from germline or somatic mutations in a single driver gene. The aim of this study was to characterize the genetic background and clinical characteristics related to genetic profile of patients with PPGLs from Romania. We retrospectively retrieved data of 125 patients consecutively registered, diagnosed with PPGLs in a tertiary referral center of endocrinology from Romania, between 1976 and 2022. We identified 88 (70.4%) women, and 37 (29.6%) men, with a mean age at diagnosis of 48.5 ± 15 years. From these 125 patients, 80 (64%) were submitted to the genomic study; 35% (n = 28) had a germline mutation (*20 RET, 3 VHL, 1 SDHB, 1 NF1, 1 SDHD, 1 FANCA, 1 CASR*) while 65% (*n* = 52) presented sporadic disease. Patients with hereditary disease had significantly lower age at diagnosis comparing to sporadic cases (37 ± 15 vs. 49.9 ± 12.2 years, *p* = 0.001). Bilateral tumors developed in twelve patients from the hereditary group. Metastatic disease was described in 4 out of 80 patients (2 of them with hereditary disease). Patients from sporadic group tended to have a right lateralisation of the tumour compared to hereditary cases, where the tumour was more often left sided. *RET* pathogenic variant (*p.Cys634Trp*) associated with MEN2A syndrome was the most prevalent in Romanian population with PPGLs and could be considered as a founder effect. Patients with hereditary disease are diagnosed at a younger age and develop bilateral tumors more frequently compared to sporadic cases.

## 1 INTRODUCTION

Pheochromocytomas and paragangliomas (PPGLs) are rare neuroendocrine tumours that originate from chromaffin cells and occur in adrenal medulla and in sympathetic or parasympathetic ganglia.^1^ Comprehensive molecular analysis revealed that PPGLs have a remarkable diversity of driver alteration including germline and somatic mutations. Despite a low incidence (0.8/100.000), over one‐third of PPGLs are associated with inheritable syndromes—the highest heritability rate among solid tumours.^2^ Currently, nearly 70% of PPGLs result from germline or somatic pathogenic variants (PVs) in a single driver gene.^3^

According to their transcriptional pattern, mutations involved in PPGLs are allocated to 3 clusters.^4^

Cluster 1 includes genes related to the Krebs cycle. Characteristic genes are those from SDHx complex (*SDHA*, *SDHAF2*, *SDHB*, *SDHC*, *SDHD*), *FH*, *MDH2*, *GOT2*, *IDH1*, *SCLC25A11*, *EPAS1* and *VHL*.^3^, ^4^ The presence of germline or somatic PVs in these genes leads directly or indirectly to dysregulation in HIF1α and HIF2α, generating pseudohypoxia, increased angiogenesis and cell proliferation.^4^, ^5^, ^6^

Cluster 2 has signature activation of MAP kinase signalling pathways. The following mutated genes are included in cluster 2: *NF1*, *RET*, *HRAS* and *TMEM127*.^4^

These mutations lead to overactivation of the phosphatidylinositol‐3‐kinase (PI3K)/AKT, mechanistic target of rapamycin (mTORC1)/p70S6 kinase (p70S6K), and RAS/RAF/ERK signalling pathways, generating overactivation of cellular growth, angiogenesis and survival.

Cluster 3 (identified by the Cancer Genome Atlas [TCGA]) is caused by dysregulation in Wnt pathways and include only somatic mutations with an increased risk of metastatic PPGLs.^6^, ^7^

Approximately 50%–60% of hereditary cases belong to cluster 1 while 40%–50% to cluster 2.^4^

A combination of particular demographic, geographical, and historical conditions has led to a diverse and specific geographical distribution of the genotype and subsequent phenotype of these tumours. For Romania, there is no report yet about the genetic landscape of these tumours.

In this study, we aim to characterize, for the first time, the genetic background and clinical characteristics related to the genetic profile of patients with PPGLs from Romania.

## 2 MATERIAL AND METHODS

### 2.1 Patients

Retrospective data of 125 patients consecutively registered, diagnosed with PPGLs in a Tertiary Referral Center of Endocrinology from Romania, between 1976 and 2022 were retrieved. The inclusion criteria was confirmation of PPGL by histopathology.

Patient data included age at diagnosis, gender, previous history and family history of PPGLs, presence of syndromic presentation, measurements of plasma and urinary normetanephrine (NMN) and metanephrine (MN), information about tumour locations and lateralisation, dimensions, histopathology and genetic testing results (germline panel by NGS or Sanger genetic screening of the RET proto‐oncogene, if the case had a typical MEN2A syndrome). Patients were diagnosed with PPGLs due to: (I) incidental finding of adrenal/extra‐adrenal tumours, and/or (II) symptoms‐based diagnosis and/or (III) genetic screening because they had first‐degree relatives with a hereditary PPGL.

### 2.2 Genetic analysis

Most of the DNA samples from peripheral blood cells were stocked in our biobank before the study begun. In some of the patients, the DNA was extracted during the study process. In total, we had the DNA from 80 patients. The rest of them were either lost to follow‐up or diagnosed before the era of genetic testing begun. All the genetic tests were performed after year 2000.

DNA was extracted from peripheral blood cells following a standard method.^8^

Patients with classic MEN2A/MEN2B phenotypes and atrisk relatives underwent direct analysis of the *RET* proto‐oncogene, and the remained samples were submitted to complete Next Generation Sequencing (NGS) in a Genetic Laboratory from Hungary. Oligonucleotide primers for the amplification of different RET exons were designed at intronic sequences flanking exons 8, 10, 11, 13, 14, 15, and 16. PCRs were performed in a final volume of 25 μL containing 20 mM Tris–HCl (pH 8.4), 50 mM KCl, 1.5 mM MgCl_2_, 0.2 mM deoxynucleotide triphosphate, 1 U Taq polymerase, 1 mM specific primers and using 100 or 200 ng of genomic DNA as input. The NGS sequencing was done using a commercial cancer panel—Trusight Hereditary Cancer Panel—targeting 113 genes from Illumina®. The analysis included identification of germline pathogenic genetic variants, copy number variations (CNV).

This panel covered genes related to PPGLs (*VHL*, *SDHA*, *SDHB*, *SDHC*, *SDHD*, *SDHAF2*, *NF1*, *MAX*, *TMEM127*, *HIF1A*, *FH*, *CDKN1B*, *MET*, *PPKAR1A*) and other non‐PPGLs related genes. Somatic DNA was extracted only in four patients, and the genetic test using NGS custom panel was performed for them. The results for these patients will be presented in Table VI.

According to the results from NGS tests, in agreement with the American College of Medical Genetics (ACMG), we classified the patients into two groups: pathogenic variant group (PV) and sporadic.^7^ In the sporadic group were also included patients with variants of unknown significance (VUS). They will be presented as Table 6. The study conforms to the Declaration of Helsinki and Good Clinical Practice guidelines. All patients were recruited under study protocols approved by the appropriate local institutional review boards or ethics committees of our centre. Informed consent for genetic analyses and use of existing clinical data was obtained from all patients.

### 2.3 Statistical analysis

All statistical analyses were performed using IBM SPSS Statistics for Windows, Version 25.0 (released 2019; IBM Corp., Armonk, NY: USA). Descriptive analysis included absolute (*n*) and relative (%) frequencies and summary measures [mean, standard deviation, median, minimum, maximum and interquartile range (IQR)]. We used Student T test, Pearson's *χ* ^2^ test or Fisher's exact test when necessary to compare proportions. Two‐sided *p* value was significant if less than 0.05.

## 3 RESULTS

In the study were included 125 patients with PPGLs with a mean age at diagnosis of 48.5 ± 15 years. Eighty‐eight (70.4%) were women and 37 (29.6%) men; median follow‐up duration was 3 (0–38) years (Table 1). From these 125 patients, 80 (64%) were submitted to the genomic study. The genetic landscape of those 80 PPGL patients submitted to the genomic study showed the following:

**TABLE 1:** General clinical data of the cohort.

Characteristics	*N* = 125
Age at diagnosis (years)	48.5 ± 15
Female (*n*, %)	88 (70.4)
PHEO/PGL (*n*, %)	115 (92.8)/10 (7.2)
Hereditary/sporadic (*n*, %)	28 (35)/52 (65)
Tumour diameter (mm)	53 ± 22
Metastatic (*n*, %)	11 (8.8)
Bilateral PHEOs (*n*, %)	12 (9.6)
Method of discovery (*n*, %)	
Symptoms	96 (74.5)
Incidentally discovered	20 (12.9)
Genetic screening	9 (6.5)
Recurrence (*n*, %)	19 (13.6)
Follow‐up duration (years)	3 (0–38)

Germline mutations were identified in 35% (*n* = 28) of the tested patients (20 *RET*, 3 *VHL*, 1 *SDHB*, 1 *NF1*, 1 *SDHD*, 1 *FANCA*, 1 *CASR*), and 65% (*n* = 52) were sporadic (Table 2).

**TABLE 2:** Comparison between hereditary versus sporadic group.

	Hereditary *N* = 28 (35%)	Sporadic *N* = 52 (65%)	*p* Value
Age at diagnosis (years ± SD)	37 ± 15	49.9 ± 12.2	**0.001**
Tumour localisation	9 Left 7 Right 12 Bilateral	29 Right 19 Left 4 PGL	NS
Tumour dimensions (mm ± SD)	53 ± 24	54 ± 27	0.989
Plasma MN levels (pg/mL)^a^	335 (30–2038)	338 (14–3365)	0.977
Plasma NMN levels (pg/mL)^a^	1093 (192–7720)	1496 (37–9808)	0.753

Abbreviation: NS, non‐significant.

^a^ Median (minimum‐maximum).

At diagnosis, patients with PV were younger compared to those from the sporadic group (37 ± 15 vs. 49.9 ± 12.2 *p* = 0.001). In PV group we identified 18 (64%) women and 10 (36%) men. In sporadic group 39 (75%) were women and 13 men (25%). All the patients from PV group presented with PHEOs while in sporadic group, 4 (7.6%) patients had PGLs (1 glomus caroticum, 1 pulmonary, 1 retroperitoneal, 1 Zuckerkandl) and 48 (92%) PHEOs (29 right, 19 left). Bilateral tumors (synchronous and metachronous) were identified in 12 patients (9.6%). All of them were hereditary cases. Metastatic disease was described in 4 (5%) out of 80 patients (2 of them with hereditary disease) (Table 3). We did not find any difference in tumour diameter or catecholamine levels in sporadic versus hereditary cases. Patients from sporadic group tended to have a right lateralisation of the tumour compared to hereditary cases, where the tumour was more often left sided, but without a significant difference between the two groups (Table 3).

**TABLE 3:** Pathogenic variants group and sporadic group – clinical data.

	RET (*n* = 20)	VHL (*n* = 3)	SDHB (*n* = 1)	SDHD (*n* = 1)	NF1 (*n* = 1)	CASR (*n* = 1)	FANCA (*n* = 1)	Sporadic (*n* = 52)
Age Mean ± SD, Gender (F, M)	36.5 ± 11.5 12F, 8M	12.3 ± 2.3 3F	36, M	56, F	39, M	57, F	53, F	49.9 ± 12.2 39F, 13M
Metastatic disease	2	0	0	0	0	0		2

Some of the patients from sporadic group had characteristics of potentially hereditary disease as described in Table 7.

The rest of 36 %, *n* = 46 from all the cohort were not tested due to lack of DNA material. Mean age for non‐tested patients was 52 ± 16 years old, 34F, 12M.

Overall, we found a 13.6% recurrence rate. Patients with pathogenic variant had a recurrence rate of 26.7 %, compared to 15.2 % of patients with a sporadic background. Clinical characteristics of each group of patients are as follows.

### 3.1 Pathogenic variant group

#### 3.1.1 Patients with RET PV (Table 4)

**TABLE 4:** Clinical characteristics and carrier status of the patients with RET pathogenic variant.

Family number + case with/without PPGLs	Nucleotide, protein	Age, Gender	Tumour characteristics	Other tumours	Years of follow‐up	Secretion pattern
1 (1/−)	*c.1900T > C*, *p.Cys634Arg* (*exon 11*)	22, F	Bilateral PHEO, metachronous	MTC metastatic	15	Mixt
2 (1/−)	*c.1902C > G*, *p.Cys634Trp* (*exon 11*)	24, F	Right PHEO	MTC	7	NA
3 (3/−)	*c.1900T > C*, *p.Cys634Arg* (*exon 11*)	43, M	Left PHEO	MTC	22	NA
	*c.1900T > C*, *p.Cys634Arg* (*exon 11*)	26, M	Bilateral PHEO metachronous	MTC, PHPTH	15	NA
	*c.1900T > C*, *p.Cys634Arg* (*exon 11*)	30, M	Right PHEO	MTC	5	NA
4 (3/−)	*c.1901G > A*, *p.Cys634Tyr* (*exon 11*)	27 M	Left PHEO	MTC	3	NA
	*c.1901G > A*, *p.Cys634Tyr* (*exon 11*)	51, M	Bilateral PHEO synchronous	MTC	4	NA
	*c.1901G > A*, *p.Cys634Tyr* (*exon 11*)	22, F	Bilateral PHEO synchronous	MTC	4	NA
5 (3/5)	*c.1852T > C*, *p.Cys618Arg* (*exon 10*)	22, M	Left PHEO	MTC, Right adrenal hyperplasia	6	NA
	*c.1852T > C*, *p.Cys618Arg* (*exon 10*)	51, F	Bilateral PHEO synchronous	MTC metastatic	3	NA
6 (2/−)	*c.1891G > T*, *p.Asp631Tyr* (*exon 11*)	51, F	Right PHEO	MTC	20	NA
7 (2/2)	*c.1902C > G*, *p.Cys634Trp* (*exon 11*)	40, M	Bilateral PHEO metastatic	MTC metastatic	31	NA
8 (1/1)	*c.1852T > C*, *p.Cys618Arg* (*exon 10*)	32, F	Left PHEO	MTC	8	NA
9 (1/2)	*c.1902C > G*, *p.Cys634Trp* (*exon 11*)	54, M	Left PHEO	MTC	2	NA
10 (1/−)	*c.1902C > G*, *p.Cys634Trp* (*exon 11*) *+ SDHA*	21 F	Right PHEO	MTC	1	NA
11 (4) (4/−)	*c.1902C > G*, *p.Cys634Trp*	46, F	Right PHEO metastatic	MTC, PHPTH, lichen amyloidosis	15	NA
12 (1) (1/3)	*c.1853G > C*, *p.Cys618Ser* (*exon 10*) *+ c.2071G > A and c.2712 C > G polymorphisms*	57, F	Right PHEO	MTC	5	NA
13 (1/1)	*c.1902C > G*, *p.Cys634Trp* (*exon 11*)	31, F	Bilateral PHEO metachronous	MTC	23	NA
14 (1/2)	*c.1902C > G*, *p.Cys634Trp* (*exon 11*)	32, F	Bilateral PHEO metachronous	MTC	8	NA
15 (1/1)	*c.1902C > G*, *p.Cys634Trp* (*exon 11*)	40, F	Bilateral PHEO metachronous	MTC	13	A

Abbreviations: MTC, medullary thyroid carcinoma; PHPTH, primary hyperparathyroidism.

Patients with *RET* PV represented 71.4% (n=20) out of patients with PV and 25% of the tested patients.

In 75% of *RET* cases (15/20), a mutation in codon 634 was identified [*p.Cys634Trp* (55.5%), *p.Cys634Arg/Tyr* 22.2%/16.6%], while 20% (4/20) patients had a mutation in codon 618 [*p.Cys618Arg* (75%), and *p.Cys618Tyr* (25%)], and one case in codon 631 (*p.Asp631Tyr*).

All patients presented with MEN2A syndrome. Twelve (60%) were women and 8 (40%) men. Mean age of diagnosis for all the group was 36.5 ± 11.5 years old. Mean age at diagnosis for patients with mutation in 634 codon was non‐significantly lower compared to patients with 618 codon mutation (34 ± 10.9 vs. 40.5 ± 8 years). The patient with mutation in codon 631 was diagnosed with PHEO at 51 years old. Nine patients developed bilateral tumours. Five of them were diagnosed due to genetic screening. In 10 patients, PHEO was the first manifestation of the syndrome, all of them with mutation in 634 codon. Two of them presented with metastatic disease. Median follow‐up duration was 8 (IQR 11.5) years.

#### 3.1.2 Patients with *VHL* (Table 5)

**TABLE 5:** Clinical characteristics and carrier status of the patients with *VHL* pathogenic variant.

Syndrome type	Nucleotide	Protein	Age, gender	Localization	Syndromic tumours	Secretion pattern
*VHL2A*	*c.245G > T*	*p.Arg82Leu*	14, F	Bilateral	Hemangioblastomas	NA
*VHL2A*	*c.245G > T*	*p.Arg82Leu*	14, F	Bilateral	Hemangioblastomas	NA
*VHL2C*	*c.482G > A*	*p.Arg161Glu*	9, F	Bilateral	No	NA

Three (3.75%) out of 80 patients representing 15% out of patients with PV had VHL (vonHippel Lindau) syndrome. Two patients had VHL2A (mother and daughter) and 1 VHL2C. Patients with VHL2A‐associated PHEO with hemangioblastomas of the nervous system. The third patient had only PHEO, without any other manifestation of the syndrome, but with a family history of hypophyseal adenoma (her mother). None of them developed renal clear cell carcinoma at a median follow‐up duration of 5 (range: 38–3) years. Mean age of diagnosis was 12.3 ± 2.3 years; all were women. All three patients had synchronous bilateral tumours. None was malignant.

One patient with VHL2A died due to complications related to VHL syndrome (hemangioblastomas, back spine tumours). The third patient was disease‐free at 10 years of follow‐up.

#### 3.1.3 NF1 c.5513_5514del, (p.Ser1838Tyrfs*23)

Patient with neurofibromatosis had 39 years old at diagnosis of a left PHEO with noradrenergic secretion pattern. He also had clinical signs of neurofibromatosis but no other tumours.

#### 3.1.4 *S* *DHB + MLH1 (VUS) c.725G > A+ c.902A > G (p.Arg242Hisp; Gln301Arg)*

One patient with *SDHB* and VUS of *MLH 1* had 36 years old at the diagnosis of a right PHEO, and no recurrence at 7 years from the diagnosis. He had no family history of PHEOs.

#### 3.1.5 CASR c.1856_1857delTT

Patient aged 57 years, with left PHEO and noradrenergic pattern. She also presented with hepatic and renal cysts. She had no family history of PHEOs or history of calcium metabolism abnormalities.

#### 3.1.6 SDHD

Patient diagnosed at 56 years old with left PHEO and noradrenergic secretion pattern had renal and splenic cysts.

#### 3.1.7 FANCA

Patient aged 53 years old was diagnosed with right PHEO with noradrenergic secretion pattern. He did not develop any other diseases related to *FANCA* gene mutations at 9 years of follow‐up, but had a daughter with hepatocarcinoma.

### 3.2 Patients from sporadic group including *VUS* patients (Tables 6 and 7)

**TABLE 6:** Clinical characteristics and carrier status of the patients with VUS.

Gene (number of patients)	Nucleotide	Protein	Gender, age	Clinical particularities	Family history
*MET* (1)	*c.4090C > T*	*p.Pro1364Ser*	M, 52	PHEO right	No
*MEN 1* (3)	*c.563C > T* (2)	*p.Pro188Leu*	M, 67	PHEO left	No
			M, 76	PHEO, right, familial hypercalciuric hypercalcemia, renal cysts	No
	*c.526G > T*	*p.Ala176Ser*	F, 46	PHEO right	No
*BRIP 1* (2)	*c.2285G > A* *c.728T > C*	*p.Arg726His* *p.Ile234Thr*	F, 47 F, 56	PHEO right PHEO right, hepatic metastases	No Colonic cancer
*PALB 2* (2)	*c.3217G > T* *c. PALB2*, *c.833_834delinsAT*	*p.Val1073Phe* *p.Leu278His*	F,43 M, 49	PHEO left, basilar, internal carotid aneurysm, carotid‐cavernous fistula PHEO right	No Pulmonary neoplasm
*BMPR1A*	*c.1215A > C*	*p.Lys405Asn*	F, 39	PHEO right	Pulmonary neoplasm
*KIF1B*	*c.692A > G*	*p.Arg18Cys*	F, 54	PHEO left, spondylitis, pyelocaliceal duplication	Sudden death at 46 y.o
*MSH 6*	*c.1474A > G*	*p.Met492Val*	F, 76	PHEO right Pulmonary adenocarcinoma (non‐smoker), multinodular goitre, renal cysts	No
*MSH 2 + ATM*	*c.1134_1136delAGA + c.1444A > C* *Somatic NF1 c.7966del* (*p.Val2656LeufsTer23*)	*p*.(*Glu378del*) *+ p.Lys482Gln*	F, 39	Neurofibromatosis phenotype	Son with café au lait signs
*MSH 3*	*c.2262A > G*	*p.Ile754Met*	F, 65	PHEO right	No
*RAD51B*	*c.52C > T*	*p.Arg18Cys*	F, 40	PHEO right	
*RB2*	*c.644C > T*	*p.Ser215Leu*	M, 49	PHEO right Idiopathic hypercalcemia	No
*ATM*	*c.5639C > T*	*p.Thr1880Met*	M, 62	PHEO right, primary hyperparathyroidism	No
*NF1 + MSH3*	*c.3461A > G + c.1721G > A*	*p.Asn1154Ile*	F, 47	PHEO left, uterine leiomyomas	No
*SDHB*	*c.487T > C*	*p.Ser163Pro*	F, 39	PHEO left	No
*BARD1 + APC*	*c.1333G > A* *c.8071A > G*	*p.Glu445Lys* *p.Asn2691Asp*	F, 64	PHEO, left, papillary thyroid carcinoma	Leukaemia, sudden death

**TABLE 7:** Clinical features of apparently hereditary PPGLs from sporadic group.

	Clinical features
Associated diseases	NEN‐G1 Vater ampulla, pulmonary hamartoma, GIST, renal tumour, primary hyperparathyroidism, pulmonary adenocarcinoma, hepatic hamartomas, renal and hepatic cysts, PTC, vertebral hemangiomas
Family history of neoplasms	Colonic neoplasm, pulmonary neoplasm, urinary bladder carcinoma, mandibular carcinoma, breast cancer

Abbreviations: GIST, Gastrointestinal stromal tumour; NEN, neuroendocrine neoplasia; PTC, papillary thyroid carcinoma,

#### 3.2.1 VUS

In patients with VUS, the mean age at diagnosis was 51.9 ± 12 years, 6M, 13F. All of them presented with PHEO, 12 right and 7 left. From those 19 patients with VUS, 4 had less than 40 years old at the time of diagnosis. In the following rows, we will present the patients with VUS and clinical aspects of hereditary disease. One patient (*MSH 6 c.1474A > G*) associated PHEO with pulmonary adenocarcinoma. One patient (*BRIP 1 c.728 T > C*) had malignant PHEO, with an aggressive pattern and family history of colonic cancer. One patient had clinical phenotype of neurofibromatosis. She had 35 years at diagnosis, and her son has clinical features of neurofibromatosis. In her case, we identified a somatic mutation of NF1 (*c.7966del*) and germline VUS of *MSH2* + *ATM c.1134_1136delAGA+ c.1444A > C*. One patient (*MEN1 c.526G > T*) associated PHEO and familial hypocalciuric hypercalcemia, and 2 other had PHEO and idiopathic hypercalcemia (*RB2 c.644C > T*), respectively, primary hyperparathyroidism (*ATM c.5639C > T*). Another patient, with PHEO, papillary thyroid carcinoma and colonic polyps, had a VUS of *BARD1* (*c.1333G > A*). Among patients with VUS, there are some likely pathogenic variants: *MEN1* *c.563C > T*; SDHA c.1937_1938insACAAAACT.

## 4 DISCUSSION

### 4.1 Discussion about genotype

To our knowledge, this study is the first to report the germline genetic profile of PPGLs from Romania. In the study were included 125 patients from a single tertiary referral centre. The percentage of patients with germline PV was 35%, similar to the last studies where the percentages ranged between 35% and 50%.^1^, ^5^, ^9^ In our cohort, the most prevalent mutation was in *RET*, followed by the *VHL* gene.

However, patients included in the study originated from the most important centre of endocrinology from Romania, where only *RET* gene is tested. MEN2A is a syndrome related to other endocrine diseases; therefore, when a patient has a suspicion of MTC, or hyperparathyroidism, he is redirected to our centre. This may have produced some referral bias resulting in a higher frequency of RET PV.

As other reports, our results confirm that there is a geographical pattern of distribution in PV. For instance, *SDHx* genes predominate among Western European population (*SDHB* in the Spanish population and *SDHD* in the Flemish population),^10^, ^11^ while the Brazilian population has a predominance in *RET* PV similar to Romania.^3^ The Chinese population, on the other hand, has fewer germline variants than Europeans.^12^, ^13^ Some reports have demonstrated that *RET* PV profile may also vary according to geographical area.^13^, ^14^, ^15^ As this is the first report of patients with PPGLs from Romania and one of the few reports with this topic from Eastern Europe, *RET* *p.Cys634Trp* variant could be considered as a founder effect for the Romanian population. Further studies should be carried out to clarify this issue. Other studies from Mediterranean basin, reporting the genetic background of MEN2A syndrome, found that the most prevalent variant was *RET* *p.Cys634Tyr* in a cohort from Spain and Italy.^15^, ^16^ While in Italy, Greece, Slovenia, Cyprus and Turkey, the most reported *RET* variant was *p.Cys634Arg*. In the Spanish cohort, RET *p.Cys634Trp* (our variant) was associated to a lower PHEO penetrance.^16^

Furthermore, in a large Brasilian cohort, *RET* *p.Cys634Gly* variant was most frequently described.^15^ Similar results as in our cohort, with a predominance of PHEO expression in *p.Cys634Trp* mutation (80% of patients with PHEO had this variant, similar to our finding), were described in a study from 2007 developed in the USA, including a large cohort of patients with MEN2A.^17^

## 5 DISCUSSION ABOUT GENOTYPE‐PHENOTYPE CORRELATIONS

### 5.1 Patients with pathogenic variants

As expected, patients with PV had a younger age at diagnosis than those with VUS or sporadic, consistent with previous reports.^12^ Female gender is predominant in this study, in keeping with previous reports, but there are also few studies indicating an equal gender balance.^16^, ^17^, ^18^

### 5.2 Patients with RET

PHEOs develop in about 50% of patients with MEN2A, usually around the age of 30–40 years.^18^, ^19^ This was also proved in our cohort, with an earlier penetrance of PHEO in codon 634 than in 618 or 631. This finding is consistent with previous studies.^20^, ^21^ It is known that approximately 60% of patients with MEN2A develop bilateral PHEOs.^1^ In our cohort, 45% of the patients presented with bilateral PHEOs, most of all with metachronous tumour. Based on this aspect, patients with *RET* PV need to be carefully followed as they can develop a second tumour, up to 20 years after the first diagnosis.^21^, ^22^ The particularity of this group of patients is one patient with mutation in 634 codon, multiple recurrences and metastatic PHEO, non‐responsive to 131‐I‐MIBG (metaiodobenylguadinine) treatment. This patient had a form of MEN2A with lichen amyloidosis.

### 5.3 Patients with VHL

In our cohort, VHL syndrome was identified in the two decades of life, earlier than any other PV identified. In the literature, the mean age at diagnosis of PHEOs with VHL syndrome is 30 years,^22^, ^23^, ^24^, ^25^ while in our cohort, the mean age at diagnosis was 12.3 ± 2.3 years.

The presence of bilateral tumours in patients with *VHL* is not an unexpected finding. Approximately 35%–60% of the patients with *VHL* have bilateral tumours. Interestingly, all of our patients with *VHL* had synchronous bilateral tumours already in paediatric age.

The two variants of *VHL* syndrome described in our cohort are considered to be rare based on population cohorts in the Genome Aggregation Database.^23^ In the literature, *p.Arg82Leu* segregated with bilateral PHEOs in families without any other manifestation of the disease and originated de novo in at least one family.^23^, ^26^, ^27^, ^28^ However, in our cohort, this variant was associated with hemangioblastomas. The third patient had an even rarer variant (*p.Arg161Glu*) which was firstly described in 1995 and was associated with multiple VHL tumours and to isolated PHEO as well.^23^, ^24^ In our case, this patient developed only PHEO.

### 5.4 Patient with NF1

He had a PV associated with the classical form of NF1. He had all the clinical signs of NF1, but no other tumours. He inherited this PV from his mother who also had a PHEO. It is reported that PHEO in NF1 PV stands out in the 4th and 5th decades of life.^29^ Our patient aged 39 years at the time of diagnosis, older than the mean age for patients with *RET* or *VHL*. Our patient did not develop malignant disease at 10 years of follow‐up. However, in the literature, at least one case of NF1 with metastases after 20 years of follow‐up is described.^27^, ^28^

### 5.5 Patient with *SDHB, SDHD, FANCA and CASR*

We found a unique association between a *SDHB* pathogenic variant and a VUS of *MLH1* in a patient with PHEO diagnosed at 36 years old; he did not develop metastatic disease at 7 years of follow‐up. However, early genetic characterization of his siblings is critical considering the *SDHB* malignancy‐associated risk.^1^

For *SDHD* and *FANCA*, we found large deletions that could not been identified with NGS. Both presented with PHEO and had no family history of PHEOs. It is known that *SDHD* mutation is more often associated with head and neck paragangliomas, and *FANCA* mutation is associated with Fanconi anaemia.^28^, ^30^ Our patient with *SDHD* developed PHEO and the other one with *FANCA* mutation was anaemia‐free.

*CASR c.1856_1857delTT* PV is a novel variant that was not earlier described in patients with PHEO based on Genome Aggregation Database^23^, ^31^ This patient had no calcium or parathormone abnormalities.

About these two mutations (FANCA and CASR), we rather think that this is a coincidental finding, as CASR and FANCA genes were never related to PPGLs.

Regarding patients with VUS, we will further discuss only cases with potentially hereditary treats.

### 5.6 *MSH 6* (*c.1474A > G*)

This variant was previously reported in patients with Lynch syndrome and in one patient with a pancreatic neuroendocrine tumour, but in any patient with PPGLs or pulmonary adenocarcinoma.^31^

### 5.7 *c.728 T > C*)

In one patient with malignant PHEO and family history of colonic cancer, we identified *BRIP 1* (*c.728 T > C*). This variant was identified in patients with breast and ovarian cancer as well as in patients with colorectal cancer, but not in patients with PPGLs.^30^, ^31^, ^32^, ^33^

### 5.8 *MSH2 + ATM* (*c.1134_1136delAGA* + *c.1444A > C*)

This association of genes was never described in patients with PPGLs. This variant of *MSH* gene was not described in any database, while this *ATM* variant was observed in individuals with breast and colon cancers and in a breast cancer study.^33^, ^34^, ^35^, ^36^, ^37^ Our patient, clinically presented with a *NF1* phenotype and somatic mutation in *NF1*.

### 5.9 *MEN1* (*c.526G > T*)

This variant was observed in some patients with MEN1 syndrome but was not described earlier in patients with PHEOs.^22^ Our patient did not have the signs of MEN1 syndrome but had familial hypocalciuric hypercalcemia. This association could be a part of a syndrome.

### 5.10 *ATM* (*c.5639C > T*)

*ATM* (*c.5639C > T*) was previously described in 1 out of 13.087 cases of breast cancer in the UK but was not related to PHEO.,^38^, ^39^ In our cohort, this variant was associated with a case of PHEO and primary hyperparathyroidism.

### 5.11 *BARD1* (*c.1333G > A*)

This alteration was reported in a Romanian breast cancer cases meeting where 1 out of 130 cases presented this variant.^38^ This variant was also reported in 2 of 1197 individuals with personal and/or family history of breast and/or ovarian cancers, but in the literature it was not associated with PHEO or papillary thyroid carcinoma, as our patient presented.^40^, ^41^

### 5.12 *RB2* (*c.644C > T*)

This variant was not previously described in the Human Genome Database.^23^ Our patient associated right PHEO with idiopathic hypercalcemia.

For patients with sporadic PHEOs, we can see that some of them developed other tumours that are suggestive for a syndrome, as well as family history of non‐PPGLs tumours. This finding suggests that the genetic landscape of PPGLs needs more exploration and that other mutations, which were not covered by the gene panel, could be involved in those patients, or that gene fusions are a reasonable variant as well.^42^, ^43^

## 6 CONCLUSION

Our study is the first report that summarized genetic landscape of patients with PPGLs from Romania. *RET* (*p.Cys634Trp*) PV associated to MEN2A syndrome is the most prevalent in Romanian population therefore this finding could be considered as a founder effect. Patients with hereditary disease were diagnosed in younger age and, presented bilateral tumors more frequently compared to sporadic cases. Most of them were women.

Among patients with sporadic disease, including VUS, there are some clinical features suggestive for a syndrome, which were not described earlier. For this category of patients, further reports, even functional testing of the genes should be applied in order to categorize them as a PV.

In summary, PPGLs have a high genetic determinism, and there is still a lot to discover about their genotype–phenotype correlations. A new entity of patients with PPGLs that have a VUS and clinical aspects of hereditary disease is up to be explored.

While every cohort report brings new characteristics for these patients, the already known classification of PPGLs will stand for new changes. Although genetic test is achieved in about 75%–80% of patients diagnosed with PPGLs, the genetic profile of these patients remains unknown in a significant percentage of cases.

## AUTHOR CONTRIBUTIONS

**Sofia‐Maria Lider‐Burciulescu:** Conceptualization (equal); data curation (equal); investigation (equal); methodology (equal); resources (equal); visualization (equal); writing – original draft (lead); writing – review and editing (equal). **Monica Gheorghiu:** Conceptualization (supporting); formal analysis (supporting); methodology (equal); supervision (equal); validation (equal); visualization (equal); writing – original draft (equal). **Elena Braha:** Conceptualization (equal); data curation (equal); investigation (equal). **Laura Semonia Stanescu:** Methodology (equal); visualization (equal). **Attila Patocs:** Resources (equal); supervision (equal); validation (equal). **Corin Badiu:** Methodology (lead); project administration (lead); resources (equal); supervision (lead); validation (lead).

## CONFLICT OF INTEREST STATEMENT

The authors declare no conflict of interest that could be perceived as prejudicing the impartiality of the reported study.

## Data Availability

Data available on request from the authors.
